# Activating Developmental Reserve Capacity Via Cognitive Training or Non-invasive Brain Stimulation: Potentials for Promoting Fronto-Parietal and Hippocampal-Striatal Network Functions in Old Age

**DOI:** 10.3389/fnagi.2017.00033

**Published:** 2017-02-23

**Authors:** Susanne Passow, Franka Thurm, Shu-Chen Li

**Affiliations:** Chair of Lifespan Developmental Neuroscience, Department of Psychology, TU DresdenDresden, Germany

**Keywords:** aging, neuronal gain control, dopamine, fronto-parietal network, hippocampal-striatal network, cognitive training, transcranial electrical stimulation

## Abstract

Existing neurocomputational and empirical data link deficient neuromodulation of the fronto-parietal and hippocampal-striatal circuitries with aging-related increase in processing noise and declines in various cognitive functions. Specifically, the theory of aging neuronal gain control postulates that aging-related suboptimal neuromodulation may attenuate neuronal gain control, which yields computational consequences on reducing the signal-to-noise-ratio of synaptic signal transmission and hampering information processing within and between cortical networks. Intervention methods such as cognitive training and non-invasive brain stimulation, e.g., transcranial direct current stimulation (tDCS), have been considered as means to buffer cognitive functions or delay cognitive decline in old age. However, to date the reported effect sizes of immediate training gains and maintenance effects of a variety of cognitive trainings are small to moderate at best; moreover, training-related transfer effects to non-trained but closely related (i.e., near-transfer) or other (i.e., far-transfer) cognitive functions are inconsistent or lacking. Similarly, although applying different tDCS protocols to reduce aging-related cognitive impairments by inducing temporary changes in cortical excitability seem somewhat promising, evidence of effects on short- and long-term plasticity is still equivocal. In this article, we will review and critically discuss existing findings of cognitive training- and stimulation-related behavioral and neural plasticity effects in the context of cognitive aging, focusing specifically on working memory and episodic memory functions, which are subserved by the fronto-parietal and hippocampal-striatal networks, respectively. Furthermore, in line with the theory of aging neuronal gain control we will highlight that developing age-specific brain stimulation protocols and the concurrent applications of tDCS during cognitive training may potentially facilitate short- and long-term cognitive and brain plasticity in old age.

## Introduction

**Figure 1:**
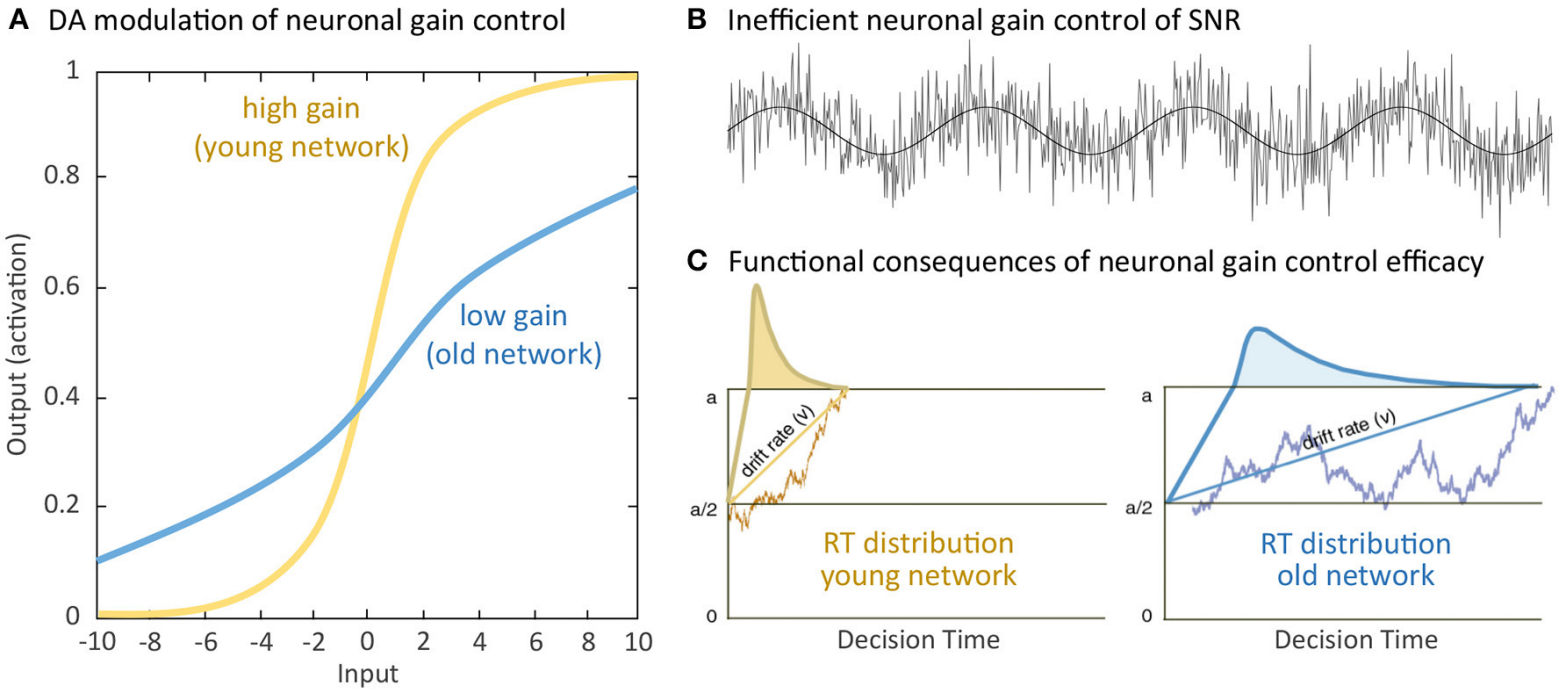
**Simulating computational effects of aging neuronal gain control: (A)** Aging-related deficiency of dopamine (DA) modulation attenuates the gain parameter of the sigmoidal transfer function relating pre-synaptic input and post-synaptic output and thus reduces the slope of the neuronal response function. **(B)** Attenuated gain control increases random processing fluctuations, which functionally reduce the signal-to-noise ratio (SNR) of information processing. **(C)** For instance, in a simple decision process between criterion a or 0, increased SNR of information processing limits the rate (drift rate, v) of evidence accumulation for either decision and precision of information processing with broader reaction time (RT) distribution, indicated by the curves, in the old compared to the young network. Further negative impacts on a wide range of cognitive functions have been discussed (Li et al., 2001; see Li and Rieckmann, 2014 for commonly observed neurocognitive aging deficits accounted for by the simulated effects of aging neuronal gain control).

Normal aging is accompanied by alterations in multiple cognitive functions with negative consequences on various daily activities. Facing the historically unprecedented global challenge of demographic change, with larger populations of individuals older than 65 years than the populations of youths younger than 20 years (Harper, 2014), a crucial agenda of geronto-psychology and geronto-neuroscience is to develop interventions that could activate the reduced but still available cognitive and brain resources in old age to buffer and delay cognitive declines. Indeed, early investigations of cognitive plasticity in the elderly provided evidence for the concept of developmental reserve capacity, which illustrates the malleability of older adults' cognitive performance been enhanced by environmental supports (Baltes et al., 1986; Baltes, 1987). Results from neurocomputational studies and empirical research provide compelling support for a close link between neuromodulation and cognitive functions. For instance, neurocomputational studies have contributed to the current understandings of cholinergic (Sarter et al., 2014), serotoninergic (Dayan and Huys, 2009) and dopaminergic (Servan-Schreiber et al., 1990; Li et al., 2001; Montague et al., 2004) systems in regulating neuronal information transmissions and their computational consequences on cognition and behavior. Of particular relevance in the context of aging, the efficacy of the cholinergic (Ellis et al., 2009; Mitsis et al., 2009; Richter et al., 2014), serotoninergic (Wong et al., 1984; Yamamoto et al., 2002; Nord et al., 2014), and dopaminergic (see Bäckman et al., 2010; Li and Rieckmann, 2014 for reviews) modulations decline substantially during the course of normal aging. The computational theory of aging neuronal gain control (Li et al., 2001) explicates a sequence of computational mechanisms that associate aging-related deficient dopaminergic neuromodulation with a variety of cognitive aging deficits. Specifically, in the simulated “old networks” deficient dopamine (DA) modulation is modeled by reducing the gain control (modeled with a lower slope) of the information transfer function that relates pre-synaptic signal input and post-synaptic response activities (Figure 1A). Consequently, the signal-to-noise ratio (SNR) of information processing is decreased in the simulated “old” network with a lower gain control, resulting in increased random processing fluctuations (Figure 1B), and consequently attenuated rate (drift rate, *v*) of evidence accumulation (Figure 1C). Generalizing from these mechanisms, other simulation studies showed that the thus simulated “old network” exhibited less distinctive representations of activation patterns and less selective recruitment of specific processing modules that accounted for aging-related declines in working memory (Li and Sikström, 2002). Furthermore, associative memory deficit (Li et al., 2005) as well as a range of other cognitive impairments commonly observed in old age could also be accounted for by the aging neuronal gain control theory (see Li and Rieckmann, 2014, for a recent review).

Notwithstanding declines in neurocognitive resources, considerable “latent reserve capacity” at the cognitive and brain levels are still preserved in old age (cf. Baltes et al., 1986), which, given appropriate environmental supports or interventions, could potentially be activated to promote successful aging (Rowe and Kahn, 1987). In particular, the concept of “developmental reserve capacity” was introduced to denote the extent that an individual's maximum cognitive performance level could be enhanced through structured environmental supports i.e., interventions (Baltes, 1987). In this context, “baseline reserve capacity” reflects the amount of available neurocognitive resources at a given moment for certain cognitive operations, whereas “developmental reserve capacity” more specifically highlights the extent of older adults' potential to benefit from interventions in raising the levels of their cognitive functions. Couched in the terms of a more recent conceptual framework of adult cognitive plasticity (Lövdén et al., 2010), activating “developmental reserve capacity” in this context denotes the potential of raising the level of organismic supplies of functional resources in older adults through interventions.

In this article, we review existing findings of cognitive training and non-invasive brain stimulation interventions i.e., transcranial direct current stimulation (tDCS) and discuss their promises and constraints in activating the reduced but still available neurocognitive resources to buffer or ameliorate older adults' cognitive functions. Furthermore, we also consider and review first promising evidence from concurrent applications of tDCS during cognitive training as means to further promote short- and long-term training effects on cognitive and brain plasticity in old age. We will discuss the potential underlying mechanisms of these positive effects within the theoretical framework of neuronal gain control, namely how cognitive training and/or brain stimulation intervention may enhance dopaminergic neurotransmission and consequently modulate the SNR of information processing with performance enhancing effects in older adults. We will focus specifically on working memory and episodic memory functions, which are supported by the fronto-parietal and hippocampal-striatal circuitries, respectively.

## Aging-related declines in fronto-parietal and hippocampal-striatal memory functions

### Aging-related declines in working memory

Cognitive control functions are described as the ability to flexibly adapt behavior by facilitating relevant over competing irrelevant information processing in order to achieve specific goals. Hence, the ability to manipulate and maintain goal-relevant information over a short period of time i.e., working memory, is essential (e.g., Engle, 2002; Cowan et al., 2005; Miller and Wallis, 2009; Fukuda et al., 2010). For instance, the content and information provided by task instructions must be actively represented and kept in mind to bias attentional allocation and response selection toward task-related goals, particularly when an inappropriate response is dominant and needs to be suppressed. Neurocognitive models of working memory suggest a dynamic interplay between prefrontal and parietal brain areas (D'Esposito, 2007; Linden, 2007; Darki and Klingberg, 2015). Posterior brain regions seem to play important roles in forming and maintaining representations, whereas prefrontal regions contribute to the selection of relevant information and the stabilization of representations during maintenance (Postle, 2006). Moreover, the fronto-striatal circuitry also implicates working memory (e.g., Cools et al., 2008; McNab and Klingberg, 2008; Darki and Klingberg, 2015). Critically, frontal and basal ganglia activity precede the filtering of irrelevant information during working memory encoding and predict storage-related parietal activity as well as inter-individual differences in working memory capacity (McNab and Klingberg, 2008).

On the neurochemical level, it has been shown that different neurotransmitters, such as serotonin (Luciana et al., 1998; Cano-Colino et al., 2014), norepinephrine (Zhang et al., 2013), and acetylcholine (Hasselmo and Stern, 2006) are involved in working memory processes (see Ellis and Nathan, 2001 for review). We focus on the role of DA here as its roles for working memory processes is best established (e.g., Sawaguchi and Goldman-Rakic, 1991; Goldman-Rakic, 1996; Arnsten, 1998; Braver and Cohen, 2000; Durstewitz et al., 2000a,b; Frank et al., 2001; Cools et al., 2008; D'Ardenne et al., 2012). Evidence from animal and human studies show that maintenance processes are supported by prefrontal DA signaling (e.g., Williams and Goldman-Rakic, 1995; Goldman-Rakic, 1996; Abi-Dargham et al., 2002). Accordingly, the dual-state theory of prefrontal DA function proposes the existence of two discrete, dynamic, and functionally different states. A D1-receptor dominated state that favors robust maintenance of information in working memory despite distractions and a D2-receptor dominated state contributing to the flexible integration of new information (Durstewitz and Seamans, 2008). Besides the role of prefrontal DA signaling in working memory processes, neurocomputational models (Braver and Cohen, 2000; Frank et al., 2001) and empirical work (D'Ardenne et al., 2012) suggest that DA signaling in the basal ganglia acts as a gating mechanism, which regulates the encoding of new information in the prefrontal cortex (PFC) and consequently the updating of context information in working memory. Selective lesions of prefrontal DA neurons in animals were associated with increased striatal DA release (Roberts et al., 1994), while enhancing DA activity in the PFC inhibited striatal DA release (Kolachana et al., 1995; Karreman and Moghaddam, 1996). Furthermore, an overexpression of D2 receptors in the striatum led to alterations in prefrontal D1 receptor activity and consequently functional impairments in working memory and behavioral flexibility tasks (Kellendonk et al., 2006). Taken together, being closely intertwined via the cortico-striato-cortical pathway the interactions between prefrontal and striatal DA systems are crucial for working memory processes and adaptive, goal-directed behavior.

There is a wealth of evidence that normal aging is accompanied by significant declines in working memory (e.g., Bopp and Verhaeghen, 2005; Borella et al., 2008; Li et al., 2008; see Lever et al., 2006; Sander et al., 2012 for reviews). At the brain functional level, aging-related changes in working memory are associated with altered task-related activations in prefrontal and posterior brain regions in older compared to younger adults (e.g., Grady et al., 1998; Cabeza et al., 2004; see Rajah and D'Esposito, 2005 for review; Rypma and D'Esposito, 2000; Schneider-Garces et al., 2010). Similarly, compared to younger adults, older adults did not show significant striatal activation during a working memory task before training intervention (Dahlin et al., 2008a). At the neurochemical level, there is ample evidence that the density of pre-synaptic (DA transporter) and post-synaptic (D1 and D2 receptors) DA markers in striatal and extra-striatal regions decline markedly from early to late adulthood (see Bäckman et al., 2010 for review). Lesion and pharmacological animal studies provide direct evidence that DA depletion (Brozoski et al., 1979; Collins et al., 1998) but also excessive DA receptor stimulation (Murphy et al., 1996; Zahrt et al., 1997) in the PFC had negative consequences for working memory functions. For instance, depletion of DA in the dorsolateral prefrontal cortex (DLPFC) in rhesus monkeys resulted in impaired working memory performance, which could be pharmacologically reversed by the DA precursor levodopa and the DA agonist apomorphine (Brozoski et al., 1979). In humans, reduced frontal and striatal DA markers were associated with an under-recruitment of the fronto-parietal network during working memory (Landau et al., 2009; Bäckman et al., 2011a) as well as reduced fronto-striatal (Klostermann et al., 2012) and fronto-parietal (Rieckmann et al., 2011) functional connectivity. Interindividual differences in caudate D1 receptor density were related to interindividual differences in functional connectivity of the right DLPFC to the right parietal cortex and of the medial PFC to the right intraparietal sulcus and postcentral gyrus during working memory performance (Rieckmann et al., 2011). In a similar vein, Klostermann et al. (2012) could show that suboptimal levels of DA synthesis capacity in the caudate were correlated with reduced functional connectivity between the right inferior frontal gyrus and the caudate, which in turn was associated with decreased working memory performance. Thus, aging-related differences in functional activations and connectivity in the cortico-striato-cortical pathway seem to be linked to suboptimal DA signaling and may underlie aging-related changes in working memory performance.

### Aging-related declines in episodic memory and spatial learning

The memory of experienced events i.e., episodic memory, encompasses multiple facets of information. For instance, the memory about a conversation includes the content of the conversation, the persons involved as well as the time and spatial location in which the conversation took place. Associative memory mechanisms are required to bind the different aspects of an experience into an integrated episode in long-term memory. The fronto-hippocampal circuitry implicates the strategic organization and elaboration of memory materials as well as the binding of different aspects of memory features during encoding, memory consolidation, and memory retrieval (Simons and Spiers, 2003), for instance pattern association which describes the function to link certain input and certain memory patterns to enable memory retrieval also with varying input patterns. Relative to semantic memory (i.e., memory for specific facts or knowledge), older adults are particularly impaired in episodic strategic organization and elaboration that are subserved by the frontal executive control processes as well as associative mechanisms that implicate the hippocampal regions (Chalfonte and Johnson, 1996; Old and Naveh-Benjamin, 2008; Shing et al., 2008). For instance, older adults' episodic memory deficit was particularly apparent in conditions requiring the memorization of associations between memory items (Naveh-Benjamin, 2000) relative to memory of single items. The aging neuronal gain control theory accounted for older adults' associative binding deficit through the less distinctive representations of the associations between items, which was the computational consequence of attenuated gain control in the memory network (Li et al., 2005). Moreover, ample evidence from functional magnetic resonance imaging (fMRI) and positron emissions tomography (PET) studies relates deficits in episodic memory encoding and retrieval in old age with alterations in functional episodic memory networks, especially with patterns of functional under-recruitment and non-selective additional bilateral recruitment of prefrontal regions, which is not observed in younger adults (see Reuter-Lorenz, 2002; Nyberg et al., 2012 for review). For instance, during episodic memory encoding older adults showed additional activation in right frontal regions while at the same time task-relevant left frontal regions were under-recruited, probably due to insufficient (i.e., non-selective) allocation of brain resources (e.g., Logan et al., 2002; Leshikar et al., 2010). Similarly, during episodic memory retrieval, older adults showed reduced selectivity of prefrontal activation during context (Cabeza et al., 2000) and recognition memory tasks (Madden et al., 1999) and reduced specificity of prefrontal and hippocampal activations during retrieval of item vs. relational memory information (Giovanello and Schacter, 2012). Simulation results from the aging neuronal gain control theory indicate that such aging-related increases of non-specific recruitments of presumably distinct processing pathways may, in part, be related to deficient DA modulation of the underlying task relevant networks (Li and Sikström, 2002).

One other specific aspect of episodic memory i.e., the spatial configuration of a memory episode, relies particularly on the hippocampal-striatal circuitry (see Moser et al., 2008 for review). Animal research showed that, whereas complex representations of spatial layouts and locations relative to environmental geometric features (e.g., spatial boundaries and shapes of the environment) are supported by the hippocampus (e.g., O'Keefe and Dostrovsky, 1971; O'Keefe and Burgess, 1996; Hartley et al., 2000), the computationally less demanding cue-based spatial learning (e.g., using fixed cue–location associations) is mainly subserved by the dorsal striatum (e.g., Packard et al., 1989; Packard and McGaugh, 1992; McDonald and White, 1994; Miyoshi et al., 2012). Applying desktop virtual reality-based fMRI spatial navigation tasks in humans, a similar dissociation was shown in healthy young adults with stronger hippocampal involvement during spatial exploration of new routes and during learning and remembering of object locations relative to a visible boundary; whereas, stronger striatal activation was shown during route following and during learning and remembering of object locations relative to an intra-environmental cue (e.g., Hartley et al., 2003; Iaria et al., 2003; Wolbers and Büchel, 2005; Doeller et al., 2008). Younger adults further showed a prioritization of relying on hippocampal-dependent spatial over striatal-dependent cue-based navigation strategies (e.g., Bohbot et al., 2012; Wiener et al., 2013). Other aspects of spatial navigation such as path integration that strongly rely on self-motion without the need of visual input also involve hippocampal-based spatial processing. Path integration, however, implicates additional human motion complex activity together with working memory-related location updating and monitoring processes of the medial PFC (e.g., Wolbers et al., 2007; De Nigris et al., 2013) and performance differences in path integration across human adulthood are, so far, not entirely understood (e.g., Harris et al., 2012, but Skolimowska et al., 2011). The complexity of the brain network underlying spatial navigation notwithstanding, we will in the following primarily focus on spatial memory subserved by the hippocampal-striatal circuitry.

Of specific interest, the relative prioritization of hippocampal- and striatal-dependent processes of spatial learning is influenced by aging. With increasing age, spatial learning, and memory decline, with an overall bias toward relying on cue-based strategies and recruitments of striatal regions (e.g., Moffat and Resnick, 2002; Driscoll et al., 2005; Bohbot et al., 2012; Etchamendy et al., 2012; Harris et al., 2012; Rodgers et al., 2012; Konishi and Bohbot, 2013; Wiener et al., 2013; Schuck et al., 2015). Specifically, whereas younger adults' behavioral data and hippocampal activity was consistent with a computational model predicting object locations relative to the geometry of the virtual environment's boundary, older adults' navigation behavior was best predicted by a model interfering object locations relative to an intra-maze location cue and was associated with larger caudate than hippocampal activation. Behaviorally, aging-related deficits in spatial learning were more prominent in hippocampal-dependent boundary learning than in striatal-dependent cue-based learning (Schuck et al., 2015). Previous research indicated that aging-related structural and neurobiological alterations in the hippocampus (see Rosenzweig and Barnes, 2003 for review; Wilson et al., 2006) as well as neuromodulatory changes in the midbrain DA system (see Bäckman et al., 2010; Li and Rieckmann, 2014 for reviews) might contribute to deficits in spatial learning and memory in old age. During normal aging, hippocampal volume progressively declines by 1–2% per year (Raz et al., 2005), which presumably affects spatial memory performance in old age (Erickson et al., 2011). Based on evidence from animal studies, the aging hippocampus, especially the perforant path receiving input from the entorhinal cortex, is further characterized by a multitude of subtle alterations in synaptic plasticity, including loss and shrinkage of synapses (Geinisman et al., 1992; Smith et al., 2000; Nicholson et al., 2004), reduced excitability leading to increasing stimulation thresholds (Barnes et al., 1994, 2000) and faster decay of long-term potentiation (Landfield et al., 1978; Barnes and McNaughton, 1985). Atrophy of the perforant path was also observed in healthy older compared to younger adults using diffusion tensor imaging (Kalus et al., 2006) and was even more pronounced in postmortem brain tissue of older adults with mild cognitive impairment (MCI) despite otherwise comparable volumes in the unimpaired and MCI groups (Scheff et al., 2006). Moreover, the extent of synaptic loss in the perforant path was negatively correlated with pre-mortem memory status. Taken together, aging-related changes in structure and function of the hippocampus may at least in part underlie older adults' increased reliance on striatal-dependent cue-based navigation strategies.

Evidence from animal research indicates that midbrain DA modulation of the hippocampus plays an important role in stabilizing transient memory traces and maintaining encoded memory associations in long-term memory (Bethus et al., 2010; see Lisman and Grace, 2005 for review; Rossato et al., 2009). In the context of spatial learning, Kentros et al. (2004) showed that DA D1/D5 agonist enhances the stability of hippocampal place fields in rats. In humans, a recent pharmacological imaging study showed that a DA agonist and DA precursor levodopa enhanced episodic memory and brain activation in older adults (Chowdhury et al., 2012). Relatedly, recent behavioral genetic evidence showed that genetic predispositions of DA transporter (*DAT1*) and receptor (*DRD2*) genes are associated with individual differences in serial memory (Li et al., 2013) and long-term episodic memory forgetting, particularly in older adults (Papenberg et al., 2013). In terms of spatial learning, a recent study with Parkinson's (PD) patients showed that, after the patients had some prior experiences with a given spatial environment, the prioritization of hippocampal-dependent boundary learning was increased relative to striatal-dependent cue-based learning when they were on dopaminergic medication (Thurm et al., 2016).

**Figure 2:**
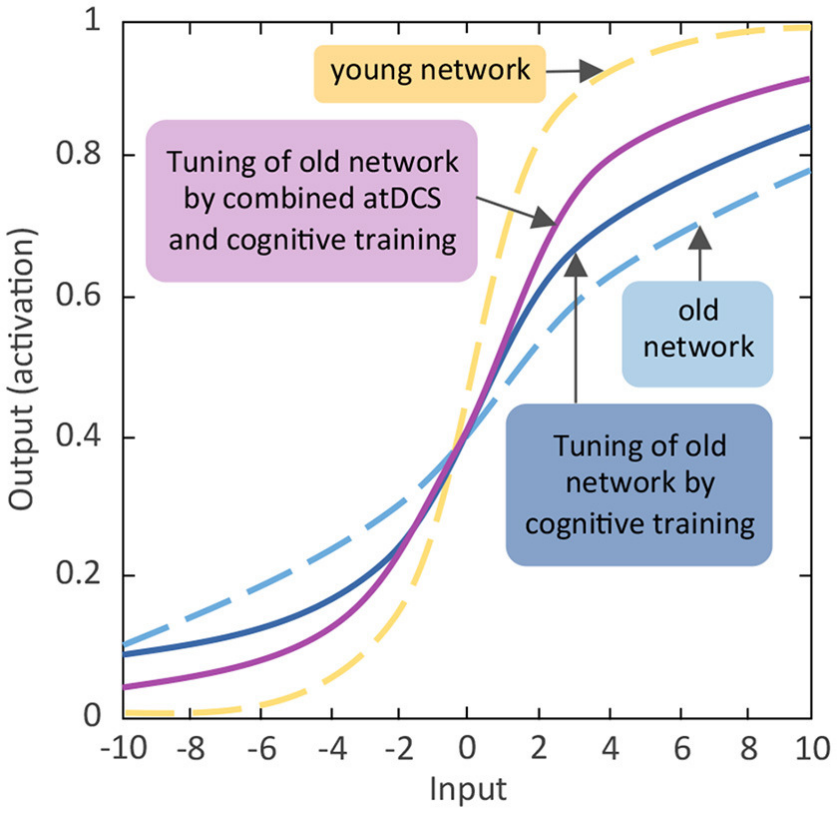
**Schematic diagram of expected effects of activating aging neuronal gain control through cognitive training and non-invasive brain stimulation**. Comparable to Figure 1A the y-axis indicates the activation value of units of the artificial neural network. The activation value as bounded by the sigmoidal activation function is between 0 and 1. The x-axis denotes incoming excitatory or inhibitory inputs, which ranged from −10 to +10. The s-shaped logistic activation function transforms the net inputs into the strength of an output signal. The responsivity of a unit to inhibitory or excitatory inputs is modulated by the slope of the function, which is regulated by the gain parameter (see Li et al., 2001). Reducing the slope flattens the activation function and the unit becomes less responsive, whereas steepening the slope of the function enhances the responsivity.

Taken together, in the two sections above we have reviewed findings indicating that normal aging is associated with prominent declines in working memory and episodic memory, with negative consequences for older adults' daily activities. Structural and functional changes as well as aging-related suboptimal dopaminergic neuromodulation in the fronto-striatal-parietal and fronto-hippocampal-striatal brain network, respectively, may contribute to these aging-related working memory and episodic memory impairments. According to the framework of the aging neuronal gain control theory (Li et al., 2001), reduced working memory and episodic memory capacity may stem from suboptimal DA modulation of the relevant networks, which may impair the SNR of information transfer within and between the respective brain circuitries, thus causing reduced specificity of information processing and less distinctive brain activation patterns. Facing increasing population aging, developing interventions that could activate the developmental reserve capacity in older adults and augment the aging brain's attenuated neuronal gain control to maintain or promote working memory and episodic memory functions (see Figure 2 for a schematic diagram) is of high societal relevance. In the following sections, evidence for why cognitive training and non-invasive brain stimulation can be seen as potential candidate interventions for promoting the aging brain's neuronal gain control will be reviewed, alongside with critical discussions about the short- and long-term effects of these interventions.

## Intervention methods enhancing neuronal gain control

### Behavioral training interventions enhancing neuronal gain control

Ameliorating older adults' cognitive decline through behavioral interventions has received a lot of attention during the last couple of years. Thus, a plethora of heterogeneous intervention methods has been developed and evaluated. For instance, cognitive, physical or combined cognitive and physical interventions (see Bamidis et al., 2014 for review) as well as action video game training (see Bavelier et al., 2012 for review) have been shown to induce behavioral and/ or brain plasticity effects. In the following we will primarily focus on cognitive training interventions in the working memory and episodic memory domain and refer readers interested in other interventions methods to the cited reviews.

Cognitive training promotes structural changes in the brain's gray and white matter. According to the animal literature, candidate cellular mechanisms underlying gray matter plasticity encompass axon sprouting, dendritic branching and synaptogenesis, neurogenesis and glial changes (see Zatorre et al., 2012 for review). Beyond these structural changes, of specific relevance in the context of this review is the evidence for training-induced changes in neurotransmitter systems. For instance, animal studies showed that motor training in rats seems to increase the expression of muscarinic acetylcholine (Ibarra et al., 1995) and DA (MacRae et al., 1987; Soiza-Reilly et al., 2004) receptors in the striatum. Spatial working memory training in monkeys has been shown to induce a reduction in the variability of firing rates across trials and a decline in cross-trial correlations of neuronal discharges, suggesting that training could lower random processing fluctuation which functionally increases the SNR of information processing and the precision of stimulus representations in PFC neurons (Qi and Constantinidis, 2012a,b). Of note, human studies using PET imaging in younger adults provide evidence for training-induced changes in striatal (Bäckman et al., 2011b) and cortical dopaminergic neuromodulation that were associated with larger working memory training gains (McNab et al., 2009). Taken these findings together, training interventions seem to be promising candidates to enhance neuronal gain control in older adults and thus promote cognitive and brain plasticity, with potential transfer effects to other functions than the trained domains. In the following, we will review in more details adult age differences in working memory and episodic memory plasticity. Other than focusing on training gains of the trained tasks, improvements in non-trained tasks closely related to working memory or episodic memory (so-called near-transfer effects), performance gains in other functional domains (so-called far-transfer effects), and stability of training- and transfer-effects (maintenance effect) will be highlighted.

#### Age differences in working memory training-induced behavioral and brain plasticity

Lifespan age differences in cognitive plasticity following training seems to vary across cognitive domains, with comparable effect sizes of immediate working memory training gains across younger and older adults (Schmiedek et al., 2010; Karbach and Verhaeghen, 2014). In contrast, near- and far-transfer effects were shown to be present in younger adults (e.g., Jaeggi et al., 2008; Chein and Morrison, 2010) but reduced or absent in older adults (e.g., Buschkuehl et al., 2008; Dahlin et al., 2008b; Li et al., 2008; Schmiedek et al., 2010; Richmond et al., 2011; Brehmer et al., 2012). With regard to maintenance effects in older adults there is evidence that training and transfer-effects of working memory training remain stable over a period of months (Dahlin et al., 2008b; Li et al., 2008; Borella et al., 2010; Richmond et al., 2011; Zinke et al., 2014).

**Figure 3:**
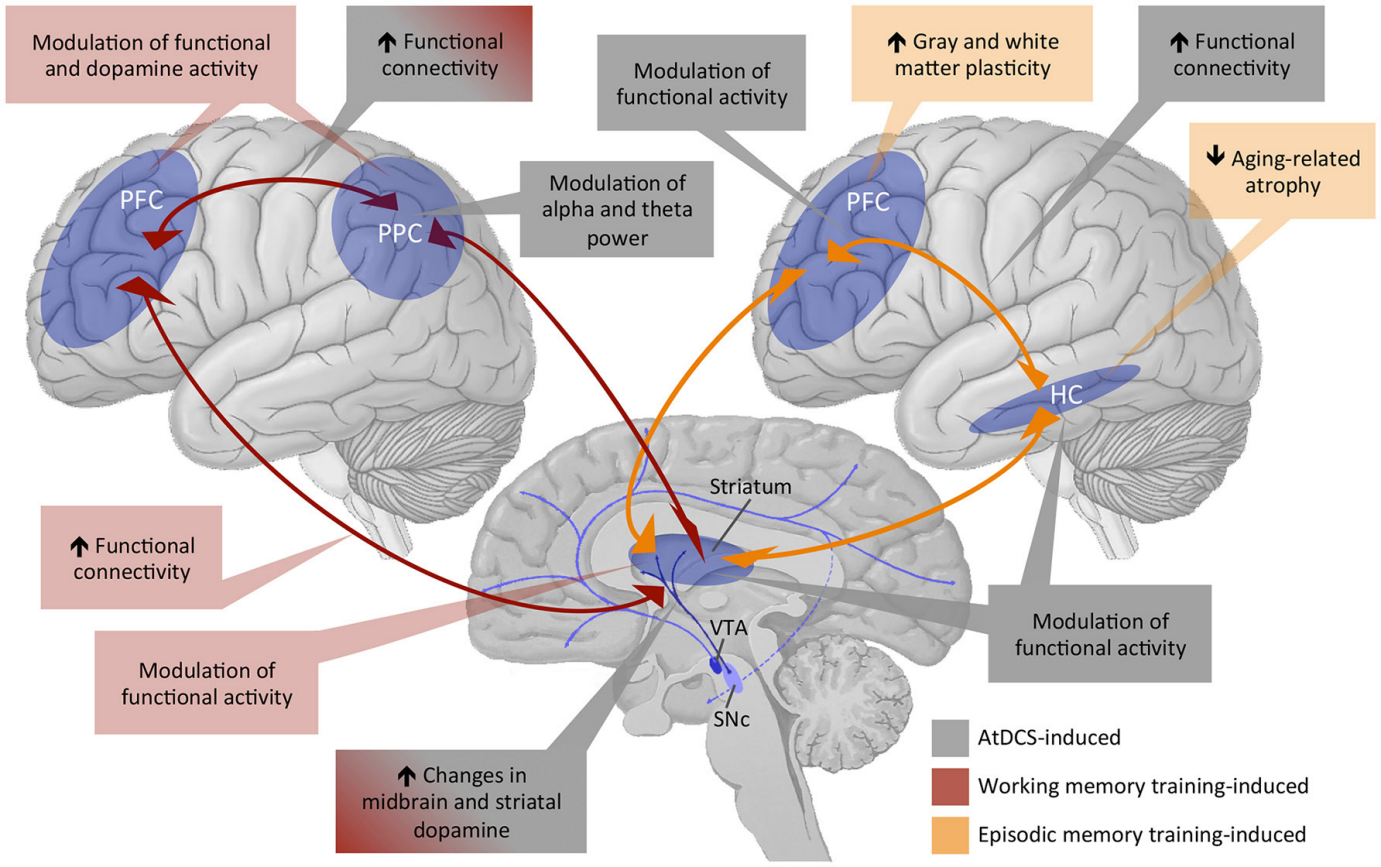
**Overview of existing evidence of training and tDCS effects on working memory and episodic memory functions subserved by the fronto-striatal-parietal and fronto-hippocampal-striatal circuitries**. PFC, prefrontal cortex; PPC, posterior parietal cortex; HC, hippocampus; VTA, ventral tegmental area; SNc, subthalamic nucleus; AtDCS, anodal transcranial direct current stimulation.

Working memory training studies in humans have revealed quantitative changes in functional activation (see Constantinidis and Klingberg, 2016 for review; Olesen et al., 2004; Dahlin et al., 2008a; Jolles et al., 2013; Kühn et al., 2013; Thompson et al., 2016) and DA signaling (McNab et al., 2009; Bäckman et al., 2011b) of the fronto-striatal-parietal network (see Figure 3 for an overview diagram). For instance, compared to pre-training fronto-parietal functional connectivity increased in younger adults (Jolles et al., 2013; Thompson et al., 2016). Furthermore, changes in striatal brain activity have also been observed and associated with working memory training-induced improvements (Dahlin et al., 2008a; Kühn et al., 2013). Of note, using PET imaging in humans, McNab and colleagues provide evidence for a training-induced enhancement in cortical DA neuromodulation that is reflected by reduced D1-receptor binding potential, which could reflect enhanced DA release after training in task-relevant brain areas. Individuals who showed greater training-induced changes in D1 receptor binding potential also showed greater training-related improvements in working memory performance (McNab et al., 2009). A further PET imaging study could show that working memory training results in enhanced striatal DA release in younger adults (Bäckman et al., 2011b).

So far, studies investigating the neural correlates of working memory training in older adults are rather scarce. There is evidence for training-induced decreases in cortical brain activations (frontal, parietal, temporal, occipital), pointing to an increase in neural efficiency, and training-induced increases in subcortical (thalamus and caudate) brain activations. Critically, the degree of the striatal changes was associated with training gains (Brehmer et al., 2011). Regarding transfer effects of working memory training, Dahlin and colleagues indicated that younger adults' transfer effects were based on training-induced increases in striatal activity in the trained and transfer task whereas this was not the case in older adults (Dahlin et al., 2008a). Thus, based on these results and given the working memory training-induced effects on striatal DA release (Bäckman et al., 2011b), aging-related reduction in transfer effects in older adults may be driven by their deficient striatal DA functioning.

#### Age differences in episodic memory training-induced behavioral and brain plasticity

Episodic memory plasticity has been shown to be more limited in old age compared to young adulthood or childhood (see Brehmer et al., 2007; Shing et al., 2010 for review; Shing et al., 2008). These age differences in training-induced plasticity are more pronounced for episodic compared to working memory (see Lindenberger, 2014 for review; Schmiedek et al., 2010). Notwithstanding the more limited episodic memory plasticity in old age, cognitive interventions might be able to reduce aging-related performance disadvantages by providing sufficient environmental support (cf. Lindenberger, 2014). For instance, aging-related under-recruitment in prefrontal regions can be reversed when encoding strategies are externally provided rather than self-initiated by the participants (Logan et al., 2002).

Early episodic memory training interventions mainly focused on instructing mnemonic (e.g., method of loci) and other memory strategies in order to facilitate task-specific encoding or retrieval in younger and older adults (see Brehmer et al., 2014 for review). For instance, Brehmer and colleagues compared the effects of a multisession mnemonic training in a lifespan sample, from childhood to old age. As a function of mnemonic instruction and adaptive training, all age groups showed improvements in the trained memory task but with older adults clearly showing the smallest training gains (Brehmer et al., 2007). Other studies showed equivocal or less promising results of various memory trainings (e.g., Jennings et al., 2005; Craik et al., 2007; Lustig and Flegal, 2008). In the very old (i.e., older adults aged 75–100 years or older), memory plasticity seems to be further reduced resulting in observable but very small negligible gains from instruction and adaptive practice compared to old adults below the age of 75 years (Singer et al., 2003). Training gains in very old age might be increased when memory training is combined with other training modules (Oswald et al., 2006) or intervention techniques.

In the COGITO study (Schmiedek et al., 2010), 100 days of memory training with verbal, numerical, and spatial material was associated with reliable near-transfer effects in both younger and older adults. However, the effect sizes for performed episodic memory tasks and latent cognitive variables were rather small in older adults (latent effect size of .09 compared to .52 in younger adults). Similarly, the ACTIVE study investigated potential far-transfer effects to functions of everyday life in older adults by comparing a verbal memory, a speed of processing, and a reasoning training with a passive control group. Cognitive training involved 10 sessions of 60–75 min over 5–6 weeks, followed by four additional training sessions two and 5 years after the initial training intervention was completed. The memory training group showed significant practice gains in the trained cognitive domain, which were stable up to 5 years after the intervention, but no further gains following additional training and no far-transfer effects of the memory training or the additional memory training on measures of everyday life functioning could be observed (Ball et al., 2002; Willis et al., 2006). Overall, the literature indicates that older adults can benefit from episodic memory training but direct training gains, so far, are much smaller compared to younger age groups and other cognitive domains. Furthermore, evident (far)-transfer effects are limited at best or lacking (cf. Noack et al., 2009, 2014).

The small behavioral effects with regard to transfer and generalizability notwithstanding, episodic memory training-induced alterations in brain structure and function have been reported (see Figure 3 for an overview diagram). For instance, at the structural level, memory training was associated with increases in cortical thickness and gray matter volume in younger, middle-aged and older adults (Engvig et al., 2010, 2012, 2014). Training-induced improvements in memory performance were further positively correlated with the extent of cortical thickness increase in the lateral orbitofrontal cortex and the right fusiform gyrus (Engvig et al., 2010) and with the extent of volume increase in the left hippocampus (Engvig et al., 2014). A further study investigated effects of a spatial memory training i.e., episodic memory training with spatial context, on cognitive and structural brain plasticity in younger and older adults. Four months of spatial memory training in a virtual zoo not only facilitated task performance but also counteracted aging-related hippocampus shrinkage up to 4 months after training in both age groups (Lövdén et al., 2012). However, training-related cortical thickening in the left paracentral lobule and precuneus were only evident in younger but not in older participants (Wenger et al., 2012), indicating that aging-related differences in training-induced structural plasticity are region-specific. Additionally, hippocampal volume prior to cognitive interventions might be one predictor of memory training outcomes in old age (Engvig et al., 2012). At the functional level, effects of episodic memory training have, so far, mainly been observed in the fronto-parietal network (Nyberg et al., 2003). After being instructed to use the method of loci as a mnemonic strategy, increased brain activities in frontal as well as occipito-parietal regions were observed in younger adults. In contrast, accompanying their reduced episodic memory plasticity as indicated by the reduced training gain, older adults did not show training-related increase in frontal activity, and only those older adults who benefited from the memory training showed increased occipito-parietal activity. Moreover, animal literature indicates that DA plays a crucial role for long-term maintenance of episodic memory training-induced effects (Rossato et al., 2009; Bethus et al., 2010), although direct evidence of enhanced DA modulation after episodic memory training is still lacking. Brain-derived neurotrophic factor (BDNF) might be one further factor modulating DA effects on episodic memory consolidation following training in rodents (Rossato et al., 2009) and spatial memory training-induced effects on cognitive and brain plasticity in adult humans (Lövdén et al., 2011).

In summary, both working memory and episodic memory training research reveal that cognitive plasticity following interventions is more limited in older adults and this is particularly so in the domain of episodic memory. So far, evidence for the transfer of training-effects to related or other cognitive processes (i.e., near- and far-transfer effects) in older adults is rare. This may reflect that solely relying on cognitive training interventions could be limited in their effects in promoting behavioral and brain plasticity in older adults (see Figure 2 for a schematic diagram). Thus, other interventions or the combination of training with other intervention methods need to be explored. Since the last 15 years transcranial electrical stimulation methods (tES) are receiving increasing attention in the field of behavioral and brain plasticity. In the following, we will briefly highlight in what ways tES, particularly anodal transcranial direct current stimulation (atDCS), may be suitable for the enhancement of neuronal gain control and thus cognitive performance in older adults. Afterwards, we will review current existing findings about the behavioral and brain plasticity effects of atDCS applications in the field of working memory and episodic memory.

### Transcranial direct current stimulation (tDCS) as a means for enhancing neuronal gain control

Transcranial direct current stimulation (tDCS) in which a constant, low intensity current (1–2 mA) is passed through two electrodes is one commonly applied stimulation mode in the field of tES techniques. Besides tDCS, tES techniques also encompass transcranial alternating current stimulation (tACS) in which a sinusoidal current is applied to modulate brain oscillatory activity and transcranial random noise stimulation (tRNS) in which current intensity and frequency vary in a random manner (see Antal and Herrmann, 2016 for review). During the last couple of years the number of published articles on tES-induced effects on cognition has increased tremendously. The endeavor of reviewing findings of all three tES methods on working memory and episodic memory functions would be beyond the scope of this article. As tDCS is the most systematically studied tES method, we limited our review on tDCS studies only.

During tDCS subthreshold changes of neuronal resting membrane potentials are induced, which alter cortical excitability and activity, dependent on the direction of the current flow. Studies of stimulating the human motor cortex have shown that anodal tDCS (atDCS) facilitates, while cathodal tDCS (ctDCS) reduces excitability. Stimulations lasting for a few seconds seems to induce solely changes in membrane potentials, while longer-lasting stimulation for a few minutes induce changes in cortical excitability, which remain stable for about 1 h or longer (see Kuo and Nitsche, 2015 for review; Nitsche and Paulus, 2000, 2001). Studies applying atDCS have shown beneficial effects on cognitive functions in young (e.g., see Brunoni and Vanderhasselt for review; Parasuraman et al., 2014; Scheldrup et al., 2014), and old age (e.g., Berryhill and Jones, 2012; see Hsu et al., 2015 for review; Flöel et al., 2012), presumably by enhancing excitability (Nitsche and Paulus, 2000, 2001), facilitating synaptic (Stagg et al., 2009; Stagg and Nitsche, 2011), neural (Islam et al., 1995) and cognitive plasticity (see Filmer et al., 2014 for review; Liebetanz et al., 2002; Flöel and Cohen, 2010), and by changing brain network connectivity (e.g., Meinzer et al., 2012; Sehm et al., 2012).

Non-invasive brain stimulation techniques seem to have a modulatory effect on dopaminergic neurotransmission (Strafella et al., 2001; Keck et al., 2002; Cho and Strafella, 2009; Tanaka et al., 2013). For instance, repetitive transcranial magnetic stimulation (rTMS) over prefrontal brain regions has been shown to induce increased extracellular DA levels in striatal (Strafella et al., 2001; Keck et al., 2002) and extra-striatal brain regions i.e., anterior cingulate and orbitofrontal cortex (Cho and Strafella, 2009). With regard to tDCS an animal study provides direct evidence for a modulatory effect of tDCS on dopaminergic neurotransmission. More specifically, extracellular DA levels in the striatum of rats increased for more than 400 min following the application of 10 min cortical ctDCS but not atDCS (Tanaka et al., 2013). Combined tDCS and drug-intervention studies further support a link between DA and tDCS-induced excitability and neuroplastic after-effects (Nitsche et al., 2006; Kuo et al., 2008; Monte-Silva et al., 2010; Fresnoza et al., 2014a,b). For instance, levodopa significantly prolongs the after-effects of tDCS applied over the motor cortex (Kuo et al., 2008), but in a non-linear, dose-dependent manner (Monte-Silva et al., 2010). More specifically, low and high dosage of levodopa abolished excitatory as well as inhibitory modulatory effects of tDCS, whereas a medium dosage turned excitatory into inhibitory plasticity and prolonged inhibitory plasticity effects. Taken together, although the exact underlying mechanisms are yet not completely understood, tDCS-induced plasticity effects seem to be partly driven by changes in the dopaminergic system. Evidence of neurocomputational, receptor imaging, and behavioral genetic studies suggests that deficient dopaminergic neurotransmission contribute to aging-related declines in working memory and episodic memory (see Li and Rieckmann for review) and older adults' reduced plasticity (Kishore et al., 2014). Consequently, tDCS interventions may be a promising tool for enhancing behavioral and neural plasticity via modulating dopaminergic signaling. Within the theoretical framework of neuronal gain control tDCS-induced improvements in dopaminergic neurotransmission are likely to enhance the gain control of the information transfer function and consequently improve the SNR of information processing in older adults resulting in higher representational distinctiveness and more selective recruitment of relevant processing modules. In terms of functional consequences this more efficient processing is likely to lead to behavioral and neural benefits in working memory and episodic memory functions. In the following two sections, we will review findings on the effects of tDCS on behavioral and brain plasticity in the domains of working memory and episodic memory (for an overview of tDCS-study characteristics see Table 1).

**Table 1:** **Overview of characteristics of working memory and episodic memory tDCS studies**.

**Authors**	**Design**	**Conditions (excluding sham)**	**tDCS set-up**				**Task sample**		
			**Anode–Cathode**	**Density (mA/cm^2^)**	**Duration (min)**		***N***	**Age**	**Female%**
**WORKING MEMORY**									
Berryhill et al., 2010	Cross-over	1	left cheek–P4	0.043	10	WM	11	25.0	45
		2	P4–left cheek	0.043	10	WM	11	25.0	45
Tseng et al., 2012	Cross-over	1	P4–left cheek	0.094	15	WM	20	22.0	65
Jones and Berryhill, 2012	Cross-over	1	left cheek–P4	0.043	10	WM	20	23.25	60
		2	P4–left cheek	0.043	10	WM	20	23.25	60
Zaehle et al., 2011	Cross-over	1	left mastoid–F3	0.029	15	WM	16	23.0–27.0	62.5
		2	F3–left mastoid	0.029	15	WM	16	23.0–27.0	62.5
Berryhill and Jones, 2012	Cross-over	1	F3–right cheek	0.043	10	WM	25	63.7	57
		2	F4–left cheek	0.043	10	WM	25	63.7	57
Nilsson et al., 2015	Cross-over	1	F3–right SO	0.029	25	WM	30	69.0 ± 7.0	46.7
		2	F3–right SO	0.057	25	WM	30	69.0 ± 7.0	46.7
**EPISODIC MEMORY**									
Manenti et al., 2013	Cross-over/between	1	left/right DLPFC/PARC–cSO	0.043		LTM	32	23.7 ± 3.2	71.9
		2	left/right DLPFC/PARC–cSO	0.043		LTM	32	67.9 ± 4.7	53.1
Sandrini et al., 2014	Between	1 (reminder)	F3–right SO	0.043	15	LTM	12	67.5 ± 2.7	66.7
		2 (no reminder)	F3–right SO	0.043	15	LTM	12	67.6 ± 4.3	66.7
Sandrini et al., 2016	Between	1	F3–right SO	0.043	15	LTM	14	68.6 ± 4.2	64.3
Smirni et al., 2015	Cross-over/between	1	shoulder–F3/F4	0.029	20	LTM	20	23.6 ± 2.3	88.9
		2	F3/F4–shoulder	0.029	20	LTM	16	24.7 ± 2.2	88.9
Zwissler et al., 2014	Between	1	F3–right shoulder	0.029	15	LTM	24	24.8 ± 2.9	62.5
		2	right shoulder–F3	0.029	15	LTM	24	24.8 ± 2.9	62.5
Jones et al., 2014 (tDCS-encoding)	Cross-over	1	P3–right cheek	0.043	15	LTM	20	23.4 ± 3.3	75
Jones et al., 2014 (tDCS-maintenance)	Cross-over	1	P3–right cheek	0.043	15	LTM	20	22.2 ± 2.5	70
Pisoni et al., 2015	Between	1	P3–P4	0.043	15	LTM	15	23.5 ± 2.6	
		2	T3–T4	0.043	15	LTM	15	23.1 ± 3.5	
Boggio et al., 2009	Cross-over	1	F3–right SO	0.057	30	LTM	10	79.1 ± 8.8	60
		2	T7–right SO	0.057	30	LTM	10	79.1 ± 8.8	60
Ferrucci et al., 2008	Cross-over	1	P3/T5-P6/T4–shoulder	0.057	15	LTM	10	75.2 ± 7.3	70
		2	shoulder–P3/T5-P6/T4	0.057	15	LTM	10	75.2 ± 7.3	70
Brunyé et al., 2014	Cross-over/between	1	T8–CP6, FC6, FT10, TP10	HDtDCS	up to 20	SM	16	20.1	0
		2	T7–CPS, FC5, FT9, TP9	HDtDCS	up to 20	SM	16	20.1	
Hampstead et al., 2014	Between	1	Pz–AF4	0.057	20	SM	8	24.6 ± 2.4	50
		2	AF4–Pz	0.057	20	SM		24.4 ± 5.1	37.5
Krishnamurthy et al., 2015	Between	1	Pz–AF4	0.057	20	rs-fMRI		19–27	33.3
		2	AF4–Pz	0.057	20	rs–fMRI		19–27	33.3
Flöel et al., 2012	Cross-over	1	P6–left SO	0.029	2	SM	20	62.1 ± 9.2	50

*cSO, contralateral supraorbital cortex; SO, supraorbital cortex; HD-tDCS, high-definition tDCS; WM, working memory; LTM, long-term memory; SM, spatial memory; rs-fMRI- resting-state functional magnetic resonance imaging; N, number of participants; PARC, parietal cortex*.

#### Effects of tDCS on working memory plasticity

As aforementioned, working memory processes rely on a broad network encompassing frontal, parietal and striatal brain regions. During the last couple of years a plethora of studies assessing tDCS effects on working memory performance in humans targeting frontal and parietal stimulation sites have been published, for instance, 10 min of ctDCS with a current intensity of 1.5 mA over the right posterior parietal cortex (PPC; P4 electrode site of the International 10–20 system) impaired working memory performance dependent on the specific working memory process that was probed. Recognition performance was impaired, whereas verbal recall of the encoded objects remained unchanged. Interestingly, atDCS did not show any effect (Berryhill et al., 2010). Inconsistent with these findings, Tseng et al. (2012) could show that 15 min of 1.5 mA atDCS but not ctDCS over the right PPC had a performance enhancing effect in a visual change-detection paradigm. There is also evidence that effects of tDCS over the right PPC were only apparent for a more challenging task and that younger adults with high working memory capacity benefited from either atDCS or ctDCS application, whereas those with low working memory capacity did not (Jones and Berryhill, 2012). In contrast, applying atDCS over the right PPC revealed that participants with low compared to those with high working memory capacity performed better in a difficult change detection task during atDCS (Tseng et al., 2012). Thus, tDCS over the posterior parietal cortex seems to modulate working memory performance, but the type and the consequences of stimulation are inconsistent. The resulting heterogeneity across studies may be due to differences in task paradigms, corresponding task difficulty and interindividual differences in baseline working memory capacity. Studies investigating the effects of atDCS over the left PFC on working memory performance (e.g., Ohn et al., 2008; Andrews et al., 2011; Zaehle et al., 2011) reported more consistent performance enhancing effects. In order to reduce heterogeneity across studies a recent meta-analysis included only non-invasive brain stimulation (NIBS) studies assessing the effects of atDCS and rTMS effects over the right, left or bilateral DLPFC on performance in n-back tasks. Critically for the current review, atDCS was shown to improve n-back performance, which was reflected by shorter reaction times, when compared to sham tDCS. This pattern of results was present across different stimulus intensities, stimulus durations, and in healthy and clinical samples (see Brunoni and Vanderhasselt, 2014 for review). Unfortunately, effect sizes in dependence of stimulation site i.e., right, left, or bilateral DLPFC, were not further discussed.

Studies investigating the underlying neuronal mechanisms of tDCS-induced effects on working memory performance are scarce (for an overview see Figure 3). Zaehle et al. (2011) studied working memory performance after a single application of 15 min 1 mA atDCS or ctDCS over the left DLPFC and the corresponding changes in oscillatory activity by using electroencephalography (EEG). The results revealed that tDCS altered working memory performance and changed the underlying neural oscillations at posterior electrode sites in a polarity-specific way (Zaehle et al., 2011). Specifically, atDCS amplified, whereas ctDCS attenuated oscillatory power in the theta and alpha bands, which are both critical for working memory processes. Local increases in alpha amplitude are related with preventing uptake of irrelevant information during working memory retention, whereas theta oscillations are thought to play an important role in the integration and organization of the different cognitive processes involved in working memory (see Sauseng et al., 2010 for review). Investigating the effects of atDCS over the left DLPFC on brain network connectivity using resting-state fMRI indicated a significant increase in functional connectivity in the default-mode and left and right fronto-parietal resting-state network (Keeser et al., 2011). The relevance of fronto-parietal functional connectivty for working memory is well established (e.g., Hampson et al., 2010; Rieckmann et al., 2011), but a direct link between tDCS-induced alterations in resting-state functional connectitvity and changes in working memory performance remains to be determined.

Evidence for enhancing effects of atDCS on cognitive functions in older adults is much more limited than in younger adults but slowly accumulating. Recent meta-analyses lend support for enhancing effects of NIBS methods on cognitive performance in older adults (Hsu et al., 2015; Summers et al., 2016). Hsu et al. (2015), for instance, considered studies examining tDCS and also TMS effects on performance across a broad variety of tasks targeting different cognitive processes (e.g., working memory, episodic memory, inhibition, error awareness). The meta-analysis revealed an overall moderate effect size (0.42). However, a systematic review and meta-analysis comparable to Brunoni and Vanderhasselt (2014) including only studies applying stimulation over the same brain area, the same working memory paradigm, and analyzing the same outcome measures in older adults is unfortunately still missing. Overall there are mixed results for atDCS-effects on working memory performance in older adults. For instance, Berryhill and Jones (2012) conducted a sham-controlled experiment with atDCS over the DLPFC before a visuo-spatial and verbal working memory task. The anode was placed either over the F3 or F4 electrode site of the 10–20 International system and 1.5 mA direct current was applied for 10 min. The results indicated that atDCS improved working memory performance independently of stimulation site. Critically, only older adults with high education levels showed the stimulation effect, which may reflect that highly educated older adults employ a different working memory strategy that can be boosted by atDCS compared to older adults with lower levels of education (Berryhill and Jones, 2012). More recently, Nilsson et al. (2015) systematically investigated the influence of atDCS over the left DLPFC on performance in an n-back task in older adults. The authors compared different current intensities (1 vs. 2 mA) and investigated the temporal development of the atDCS effect i.e., n-back performance was assessed before, three times during, 5 and 30 min after the 25 min-stimulation period. The results revealed no significant effects of atDCS. Compared to sham stimulation atDCS did not modulate working memory performance at any point during or after stimulation (Nilsson et al., 2015). These results should be interpreted with caution, as possible practice effects due to multiple testing in sham and atDCS stimulation conditions may have masked the stimulation effects. However, the lack of a robust effect after a singular application of tDCS is consistent with a meta-analysis, indicating that multi-session stimulations are more effective than single-session stimulations in older adults (Hsu et al., 2015).

#### Effects of tDCS on episodic memory plasticity

Most studies investigating potential facilitating effects of atDCS on episodic memory functions focused on verbal and visual memory, which are memory functions subserved by a broader fronto-hippocampal-parietal circuitry. As direct stimulation of critical subcortical structures such as the hippocampus or striatum is not applicable in healthy human subjects, network activations via the stimulation of cortical areas as the frontal and parietal cortex are commonly applied. There is evidence indicating that, relative to sham or control site conditions, atDCS stimulation of the left DLPFC with a current of 1–2 mA for up to 20 min during or immediately after encoding of the stimulus material improved immediate recognition and retrieval or reduced long-term forgetting of verbal and visual episodic memories in younger (e.g., Javadi and Walsh, 2012; Manenti et al., 2013; Gray et al., 2015) and older adults (e.g., Manenti et al., 2013; Sandrini et al., 2014, 2016). Stimulation effects were independent of stimulation hemisphere in young adulthood but memory improvements in older adults were only observed following left hemisphere stimulation (Manenti et al., 2013). Nevertheless, beneficial stimulation effects have also been observed 48 h later (Sandrini et al., 2014, 2016) or up to 1 month after applying atDCS (Sandrini et al., 2014) in older adults. However, there are also other studies that failed to replicate these results in younger adults (Smirni et al., 2015) or even reported an increase of false alarm rates in episodic memory (Zwissler et al., 2014).

Fewer studies involving younger adults investigated potential effects of atDCS over the temporal or parietal cortices. For instance, Jones and colleagues showed facilitations in verbal long-term memory in younger adults when atDCS was administered during encoding but not during maintenance over the left PPC with a current of 1.5 mA for 15 min (Jones et al., 2014). Bilateral atDCS (i.e., the anode over the left and the cathode over the right temporal cortex or the PPC) during the recognition phase of a word list learning task showed differential effects in younger adults: recognition performance of old (hit) but not new items (correct rejection) was improved in the temporal cortex stimulation group whereas recognition performance of new but not old items was improved in the PPC stimulation group (Pisoni et al., 2015). Such findings indicate that potential effects of facilitation vs. inhibition of new afferent information depend on the stimulation site and, hence, on the underlying brain circuitry of the respective cognitive domain. Evidence from healthy aging studies is, so far, missing, but improvements in recognition memory up to 4 weeks after stimulation (over the left DLPFC or bilateral over temporoparietal areas with 1.5–2 mA for 15–30 min) had been observed in Alzheimer's disease patients (Ferrucci et al., 2008; Boggio et al., 2009).

Thus, far, there are even fewer studies, which investigated effects of tDCS on spatial learning and memory. Nevertheless, the available results offer some optimism regarding tDCS-induced spatial memory plasticity in the adult lifespan. In younger adults, applying atDCS at 2 mA for 20 min over the right centrotemporal cortex during spatial navigation in a virtual environment facilitated later performance in a sketch map drawing test that required the participants to re-draw the layout of the virtual environment from memory. Interindividual differences in the sense of direction predicted atDCS-induced spatial navigation benefits, with low-performing individuals benefitting more (Brunyé et al., 2014).

Regarding the underlying neural correlates, animal literature, so far, provides only tentative evidence that BDNF and neurogenesis in the dentate gyrus might play a role in atDCS-induced improvements in episodic and spatial memory performance (see Bennabi et al., 2014 for review). Recent studies combining tDCS with fMRI investigated the effects of tDCS on activation and functional connectivity within the fronto-hippocampal-striatal network in humans (Hampstead et al., 2014; Krishnamurthy et al., 2015; see Figure 3 for an overview diagram). Network-modulatory effects of tDCS were investigated by applying 20 min of 2 mA tDCS offline before the participants performed a spatial navigation task assessing hippocampal- vs. striatal-based spatial memory in the MR scanner. The anode and cathode were placed over midline parietal and frontal regions, respectively. The parietal-anode/frontal-cathode montage had no effect on hippocampal activity in both hippocampal- and striatal-dependent spatial navigation conditions but was associated with increased right caudate activation during sequential stimulus-response-based spatial navigation and greater connectivity between the left prefrontal and the parietal cortex. In contrast, the frontal-anode/parietal-cathode montage was associated with increased right hippocampal and bilateral activity in prefrontal regions during hippocampus-dependent navigation and with greater connectivity between prefrontal regions and the right hippocampus (Hampstead et al., 2014). The parietal-anode/frontal-cathode montage was further associated with increased fMRI resting-state functional connectivity between the superior parietal lobule and other brain regions of the spatial learning and memory network 10 min after the stimulation (Krishnamurthy et al., 2015). To our knowledge, only one study has, so far, investigated effects of atDCS on spatial memory in healthy older adults (Flöel et al., 2012). In a 2-sessions within-subject cross-over design, atDCS with a current of 1 mA for 20 min over the right temporoparietal cortex was applied during the learning phase of an object-location learning paradigm. Despite lacking tDCS-induced effects during learning, healthy older adults showed tDCS-induced benefits of memory recall 1 week after stimulation, indicating that tDCS can have medium- to long-term effects on spatial memory even in old age.

Taken together, only a small number of studies have investigated the effects of atDCS on episodic and spatial memory in older adults and existing findings indicate further needs of systematic investigations. Of note, in the domain of working memory the results are rather mixed. Two possible factors may explain the, for now, inconsistent results. Given that interindividual variability in widespread changes in brain physiology and brain plasticity increase with old age, optimal tDCS parameters (i.e., current intensity, stimulation duration, and frequency, electrode montage) for applications in older adults can be expected to differ from those for younger adults (Zimerman and Hummel, 2010; Fertonani et al., 2014). Thus, developing age-appropriate stimulation protocols require more systematic investigations. Furthermore, across various cognitive functions multi-session tDCS applications seem to be more efficient compared to single-session application in older adults (Hsu et al., 2015). Thus, tDCS applied in combination with cognitive training over multiple sessions may provide the added neural boost for enhancing and prolonging transfer effects that are known to be reduced or lacking in older adults (see Figure 2 for a schematic diagram).

## Combining cognitive training and tDCS

Very recently, a few studies have started to explore the effects of combining motor learning (Reis et al., 2009) or cognitive training with atDCS interventions in younger (Meinzer et al., 2014; Richmond et al., 2014; Au et al., 2016; Looi et al., 2016; Mancuso et al., 2016) and older adults (Jones et al., 2015b; Stephens and Berryhill, 2016). In younger adults, first evidence for atDCS-enhancing effects on training gains have been shown across various cognitive functions, e.g., arithmetic operations (Looi et al., 2016), language (Meinzer et al., 2014), and working memory (Richmond et al., 2014; Au et al., 2016). For episodic memory though, there is yet no study investigating synergistic effects of atDCS and episodic memory training neither in healthy young nor older populations. There is, to the best of our knowledge, only one study that applied 2 mA atDCS for 25 min over the left DLPFC during 10 sessions of memory training in Alzheimer's disease patients without being able to show ameliorating effects of atDCS on the training-related memory improvements (Cotelli et al., 2014).

With respect to working memory, Richmond et al. (2014) let their participants take part in an adaptive training over 10 sessions concurrent with either 15 min of 1.5 mA atDCS or sham stimulation over the left DLPFC. The results showed that compared to sham stimulation atDCS enhanced learning and near-transfer to other non-trained working memory tasks. Far-transfer or maintenance effects were not investigated. Enhanced training performance due to additional atDCS could also be reported by Au et al. (2016). In seven sessions participants received 25 min of 2 mA atDCS over the right or left DLPFC concurrent with a visual-spatial working memory training. Near-transfer to non-trained visual or spatial working memory tasks could also be observed but only in the right DLPFC stimulation group which is in line with the right-hemispheric dominance of the DLPFC for spatial working memory functions (Wager and Smith, 2003). Critically, the authors also assessed maintenance effects and could show that the atDCS- enhanced training effects remained stable up to 8 months after training completion (Au et al., 2016). Thus, there is promising evidence for prefrontal atDCS-enhancing effects on immediate training gains and near-transfer effects. The effects on far-transfer effects still need to be explored. Taken together, the currently existing empirical findings in younger adults lend support to the idea that concurrent atDCS-training applications might bolster older adults' limited working memory training and transfer gains.

There is already some preliminary but promising evidence suggesting that older adults can benefit from combined atDCS and training interventions. For instance, older participants who received 30 min of 2 mA atDCS over the DLPFC during 10 sessions of computer-based cognitive training showed greater improvements in verbal working memory compared to a sham stimulation group. This effect maintained up to 28 days (Park et al., 2014). Near- and far-transfer effects were not assessed. Jones et al. (2015b) could provide evidence for maintenance effects of atDCS on training-related improvements and transfer effects in older adults. In their study older adults received sham or atDCS over the right DLPFC, parietal, or alternating prefrontal/parietal cortices (stimulation site was varied across training sessions). The participants were randomly assigned to one of the four groups and were matched according to age, education and cognitive status. In 10 sessions, after 10 min of 1.5 mA tDCS participants performed a working memory task. All groups benefited from working memory training and showed significant improvements in the trained and near-transfer tasks. Critically, after 1 month of no contact, only the participants in the atDCS group maintained the significant improvement for the trained and near-transfer tasks. Interestingly, the magnitude of this improvement did not vary as a function of stimulation site condition indicating that all stimulation sites equally well targeted the fronto-parietal network, which could also be confirmed by current modeling (Jones et al., 2015b). In a more recent study of the same group, standard far-transfer effects (i.e., processing speed, cognitive flexibility, arithmetic) and ecologically valid far-transfer effects were assessed to investigate translation to other cognitive abilities and daily activities as e.g., scheduling appointments, driving, safety awareness, and route planning. In this study older adults took part in a 5-day working memory training combined with 15 min of either sham, 1, or 2 mA atDCS over the right DLPFC. Comparable to their first study, the authors replicated the general improvement in the trained task across all groups. Critically, 2 mA atDCS induced significantly greater far-transfer gains after 1 month of no contact (Stephens and Berryhill, 2016). Taken together, working memory training when combined with atDCS seems to offer promise in enhancing and maintaining older adults's working memory training as well as near- and far-transfer gains. Whether the effect sizes of atDCS-enhancing training gains in older adults are comparable to those of younger adults needs to be determined.

## Limitations and outlook

Notwithstanding the promising effects of combined atDCS and cognitive training interventions, there are several open questions and limitations that should be addressed in future studies. As there was no comparison group in all the combined atDCS and cognitive training studies that underwent only tDCS, no firm conclusions about the synergistic effects of brain stimulation and cognitive training intervention can yet be drawn. Thus, future work should include a tDCS-only control group to clarify whether tDCS, cognitive training and the combination of tDCS and cognitive training contribute differently to short- and long-term benefits. The response to tDCS has been shown to be state-dependent and critically vary as a function of interindividual differences in educational level (Berryhill and Jones, 2012) or baseline task performance (Jones and Berryhill, 2012; Tseng et al., 2012). It is likely that tDCS interacts with individual endogenous activity levels within the region of targeted neurons, rather than exerting a homogeneous effect across individuals (Learmonth et al., 2015). Further, the results of a previous study could show that tDCS effects were boosted after supplying a task strategy or financial motivation (Jones et al., 2015a). Thus, considerable attention should be paid to the thorough assessment of baseline task ability and the influences of motivational factors when designing future tDCS and combined tDCS and training interventions. Given the existence of an inverted-U relationship between DA level and cognition (Li and Sikström, 2002; Cools and D'Esposito, 2011) and non-linear, dose-dependent effects of levodopa on tDCS-induced plasticity (Monte-Silva et al., 2010), atDCS could also shift performance beyond the optimal range. Thus, interindividual differences in baseline DA-level should be kept in mind when interpreting interindividual differences in tDCS-induced effects. Furthermore, so far, we can only infer that the effects of combined tDCS and training interventions on working memory may be mediated through the strengthening of functional connectivity in the fronto-striatal-parietal as well as dopaminergic modulation of this circuitry (Jones et al., 2015b). Future fMRI and PET studies should, therefore, investigate the underlying neuronal mechanisms of combined tDCS and training effects in order to explore whether these effects go beyond the known training-induced changes in brain activation and functional connectivity in the fronto-striatal-parietal network (e.g., Dahlin et al., 2008a; Jolles et al., 2013; Kühn et al., 2013; Thompson et al., 2016) as well as cortical and striatal DA signaling (McNab et al., 2009; Bäckman et al., 2011b). Given that older adults' reduced cognitive plasticity following cognitive training interventions is particularly limited in the domain of episodic memory, future studies should investigate whether similarly promising results can be shown for episodic memory and maybe other cognitive domains. Furthermore, as older adults are particularly limited in transfer effects of cognitive training interventions, future work should by default include both, near- and far-transfer tasks and invest more effort in developing protocols that enable the investigation of transfer particularly to daily activities. Related to this, future work should also focus on the home-based applicability of combined tDCS and training interventions to pave ways for more ecologically valid interventions that may promote the maintenance of autonomy and quality of life in old age.

## Author contributions

SP, FT, and SL did substantial contributions to the conception and design of the review article. SP, FT, and SL drafted the work and revised it critically for important intellectual content. SP, FT, and SL did the final approval of the version, to be published. Finally SP, FT, and SL agree to be accountable for all aspects of the work in ensuring that questions related to the accuracy or integrity of any part of the work are appropriately investigated and resolved.

### Conflict of interest statement

The authors declare that the research was conducted in the absence of any commercial or financial relationships that could be construed as a potential conflict of interest. The reviewer YS declared a shared secondary affiliation, though no other existing collaboration, with one of the authors SL to the handling Editor, who ensured that the process nevertheless met the standards of a fair and objective review.
